# Associations of human papillomavirus genotypes and cervical vascular abnormality in a cohort of women underwent colposcopy, a retrospective study of 6716 patients

**DOI:** 10.3389/fonc.2023.1105482

**Published:** 2023-04-05

**Authors:** Yulong Zhang, Haibo Li, Xiaowen Li, Zhelong Li, Qianru You, Huan Yi, Yanzhao Su, Xiangqin Zheng, Yusha Chen, Jiancui Chen

**Affiliations:** ^1^ Department of Gynecology, Fujian Maternity and Child Health Hospital College of Clinical Medical for Obstetrics & Gynecology and Pediatrics, Fujian Medical University, Fuzhou, China; ^2^ Division of Birth Cohort Study, Fujian Maternity and Child Health Hospital, College of Clinical Medicine for Obstetrics & Gynecology and Pediatrics, Fujian Medical University, Fuzhou, China; ^3^ Cervical Disease Diagnosis and Treatment Health Center, Fujian Maternity and Child Health Hospital College of Clinical Medical for Obstetrics & Gynecology and Pediatrics, Fujian Medical University, Fuzhou, China

**Keywords:** human papillomavirus (HPV), cervical vascular abnormality, cervical lesions, cervical cancer, retrospective study

## Abstract

**Aims:**

Abnormal vessel patterns are specific signs in patients with early cervical abnormality and cervical cancer(CC) by colposcopy, but the impact of human papillomavirus (HPV) infections on abnormal vessel patterns remains unknown.

**Methods:**

A total of 6716 female patients with HPV infections or cytological abnormalities who underwent a colposcopy following abnormal CC screening results were included in the study. The final pathological diagnosis was confirmed to be the most severe pathological grade across cervical biopsy, endocervical canal curettage (ECC) and conization. Univariate and multivariate logistic regression analyses were used to investigate the association between HPV infections and abnormal vessel patterns, adjusting for age, gravidity and parity.

**Results:**

There were 6124 normal vascular cases by colposcopy and 592 cases with cervical vascular abnormality. The prevalence of HPV infections was 4284 (70%) in normal patients, and the prevalence of HPV infections was 479 (80%) in cervical vascular abnormality patients. HPV high-risk type 16 infection alone increased the risk of cervical heteromorphic blood vessels (aOR=3.66, 95%CI: 2.54~5.27). HPV 16 and 33 alone (other than the commonly recognized subtype of 18) or coinfection of these two genotypes could increase the risk of cervical punctate vascular and cervical vascular mosaic features and abnormal cervical blood vessels. An increased risk of abnormal cervical lesions was observed for HPV 16 and 33 alone or combined in coinfection compared to the negative group. The risk of cervical vascular abnormality was increased 10-fold by coinfection with HPV 16 and 33 (aOR=10.67, 95% CI: 4.54~25.09, P<0.001). HPV 16, 33 alone or combined in coinfection were associated with an increased risk of lesions more advanced than high-grade squamous intraepithelial lesion (HSIL) when compared to the negative group. The risk of lesions more advanced than HSIL was up to 26-fold higher in the coinfection with HPV 16 and 33 group than in the negative group (aOR=26.23, 95%CI: 11.23~61.27, P<0.001).

**Conclusion:**

HPV16 and 33 are the most dangerous HPV genotypes correlated with abnormal vascular patterns. Combined HPV16 and HPV33 infection increases the risk of abnormal vascular patterns. Combined HPV16 and HPV33 infection increases the risk of developing HSIL+.

## 1 Background

CC is the fourth most common malignancy affecting women’s health worldwide, and there are approximately 600,000 new cases and 300,000 deaths every year (1–3). HPV is the most common infection of the female genital tract and is one of the most common pathogens of globally transmitted infections (4). Genital HPV types can be grouped as high-risk (HR, oncogenic) and low-risk (nononcogenic). Low-risk or nononcogenic HPV types include types 6, 11, 42, 43 and 81, while high-risk or oncogenic HPV types include types 16, 18, 31, 33, 35, 39, 45, 51, 52, 53, 56, 58, 59, 66, 68, 73, 82 and 83 (5). Persistent HPV infection is considered to be the main cause of cervical intraepithelial neoplasia (CIN) and CC (6).

Early-stage CC or high-grade CIN is always curable. However, recurrent or metastatic CC with the feature of tumour angiogenesis has a relatively poor prognosis with a 5-year survival rate of less than 20% (7). Persistent local infection by HR-HPV, especially subtypes 16 and 18, ends at angiogenesis in the cervix, which leads to local tumour promotion and metastasis. As HR-HPV infection seems to directly activate VEGF and lead to angiogenesis in local tumours by inhibiting P53 and stabilizing HIF-1α, abnormal vessel patterns in the cervix as detected by colposcopy play a dominant role in the diagnosis and therapy of CIN and CC (7, 8). Additionally, despite the close association between HPV and angiogenesis, there have been different outcomes, and some studies have shown that only a small percentage of patients infected with HPV will develop CIN and CC (9). Therefore, many environmental factors after HPV infection, such as cervical angiogenesis and cervical histological abnormalities, must play an important role in the occurrence and progression of CIN and CC (9–13).

The impact of different genotypes of HPV infections on cervical vascular lesions in different races and regions remains unclear. There is currently no consensus on the topic of HPV infections in cervical angiogenesis and CC. Importantly, it is urgent to study the relationship between the subtype of HR-HPV and cervical vascular lesions based on large real-world sample data. We can determine the type of HPV infection in patients through HPV testing. We found that changes in cervical vascular morphology can be observed in colposcopy, and these changes were correlated with CIN and CC. Therefore, we intend to study the relationship between HPV infection and cervical blood vessel features in a large sample from southeastern China to promote progress in CC prevention, diagnosis and treatment.

## 2 Materials and methods

### 2.1 Study population

**Figure 1:**
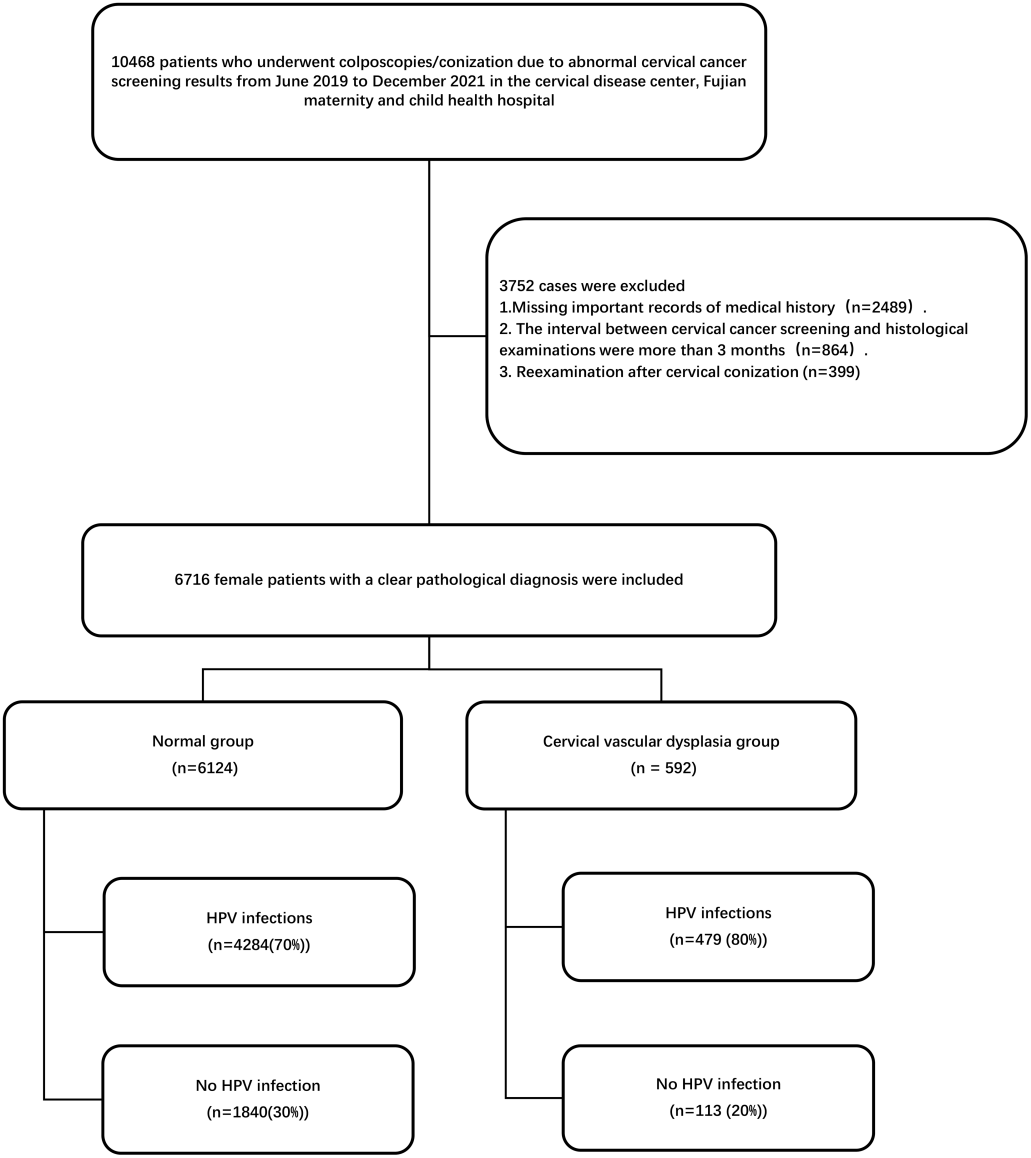
Study design. HPV, human papillomavirus.

We conducted a cross-sectional study in which patients who underwent colposcopies/conization from June 2019 to December 2021 due to abnormal CC screening results in the Cervical Disease Centre, Fujian Maternity and Child Health Hospital, were included. The CC screening consisted of ThinPrep Cytology Test (TCT) and/or HPV genotyping. In accordance with the system in the 2001 Bethesda system, the following TCT results were defined as abnormal conditions: atypical squamous cells of unknown significance (ASC-US), low-grade squamous intraepithelial lesion (LSIL), HSIL, atypical glandular cells (AGC), endocervical adenocarcinoma *in situ* (AIS), squamous cell carcinoma (SCC) and adenocarcinoma. In all cases, the time between CC screening and histological examinations was less than 3 months. The inclusion and exclusion criteria are detailed in the flowchart (**Figure 1**). Clinical information, including age, parity, gravidity, HPV genotypes, and cervical pathology, was obtained from the medical records of the department.

The study was performed according to the Declaration of Helsinki (as revised in 2013) after approval was granted by the Ethics Committee of Fujian Maternity and Child Health Hospital, Affiliated Hospital of Fujian Medical University (2022YJ002). The study was conducted without informed consent because it was a retrospective study.

### 2.2 HPV Genotyping

PCR-RDB HPV genotyping (Yaneng Biotech) was used to distinguish 18 types of HR-HPV (16, 18, 31, 33, 35, 39, 45, 51, 52, 53, 56, 58, 59, 66, 68, 73, 82, and 83) and 5 types of low-risk HPV (LR-HPV) (6, 11, 42, 43, and 81).

### 2.2 Pathological diagnosis

All patients in this study were referred for colposcopy and underwent colposcopy cervical biopsy according to the indication of the ASCCP guidelines. ECC should be carried out simultaneously when patients have the following conditions: HPV16/18 infections, AGC/AIS/HSIL cytology, and type 3 cervical transformation zone. If the results of liquid-based cytology were HSIL, AGC-FN (atypical glandular cell, favour neoplastic), or AIS, or if cervical pathological biopsy/ECC results indicated CIN2-3 (cervical intraepithelial neoplasia 2-3), cervical cone resection was also performed. Pathological evaluation of cervical biopsies/ECC and conization tissues were independently analysed by two blinded senior pathologists. The most advanced lesions among the cervical biopsies, ECC and conization tissues were taken as the final pathological diagnosis. According to the 2014 WHO classification of tumours of the female reproductive organs (4th Edition) (14) and Lower Anogenital Squamous Terminology (LAST) recommendations (15), the histologic endpoints were defined as follows: normal cervix; LSIL, which includes CIN1- and p16-negative CIN2; HSIL, including p16-positive CIN2 and CIN3; AIS; and invasive CC. Furthermore, HSIL, AIS and invasive CC were classified as HSIL+.

### 2.3 Cervical vascular abnormality and procedure for colposcopic examination

**Figure 2:**
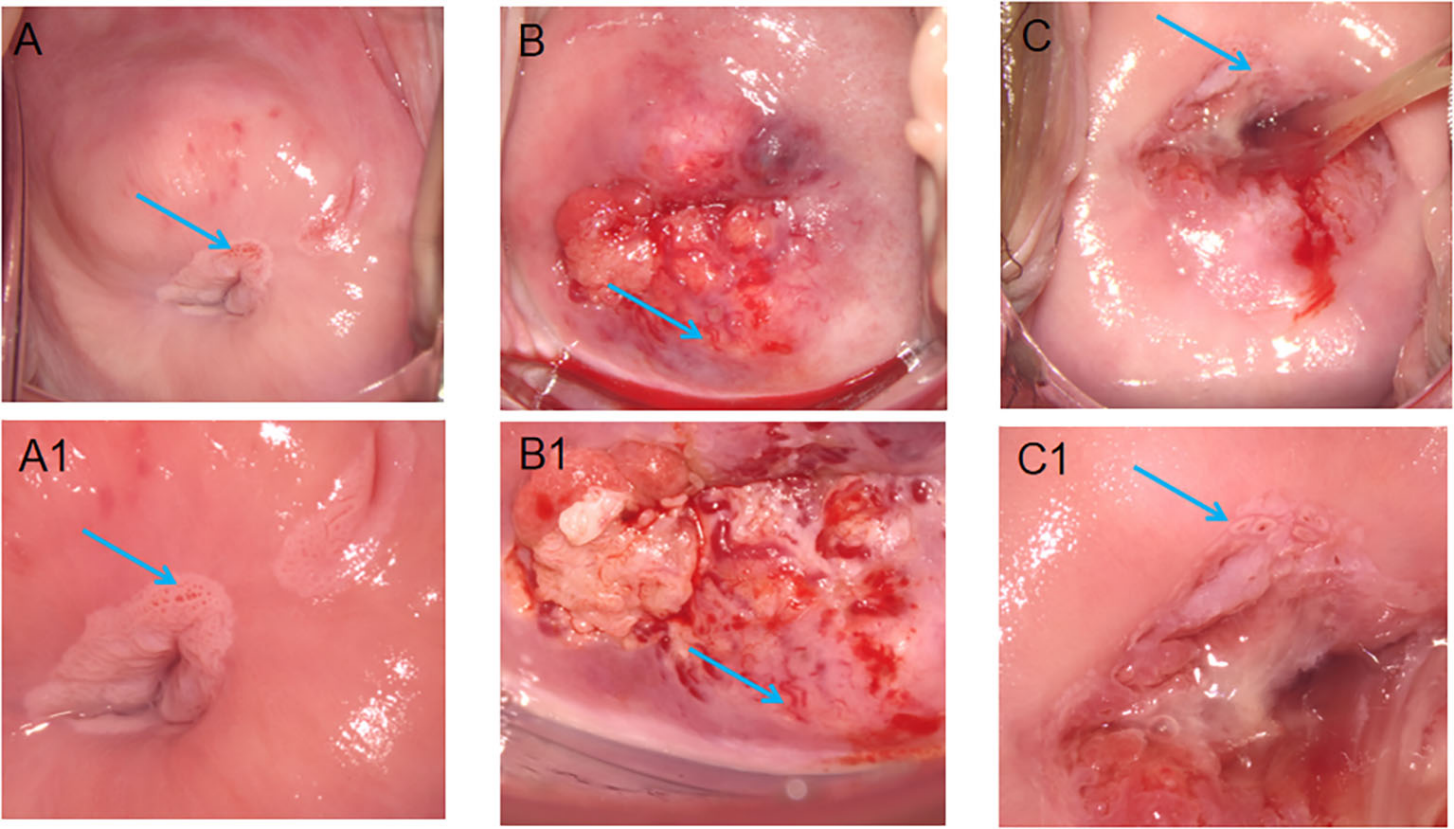
Features of different cervical vessels on colposcopy. **(A)** The arrows point to the punctate vessels of the cervix; **(B)** The arrows point to the cervical heteromorphic blood vessel; **(C)** The arrows point to the cervical vascular mosaic; **(A1, B1, C1)** is the image after **(A–C)** is enlarged, respectively.

Cervical vascular abnormality referred to lesions that were demonstrated as punctate vascular, cervical vascular mosaic, cervical heteromorphic blood vessel (**Figure 2**). Leisegang D-10625, Model1DS Ur Nr 55764, Colposcope, Berlin, Germany, was used to examine the cervix. The cervix was exposed for examination through an appropriately sized Coscus speculum, which was used to expose the vagina in the lithotomy position. We with 0.9% normal saline to dislodge excess mucus discharge before the application of 3% acetic acid. Colposcopic abnormalities were divided into normal, abnormal or unsatisfactory. Punch cervical biopsy tissue forceps were applied to the abnormal areas for biopsy. The cervical specimens were processed in the histopathology laboratory. All the specimens were diagnosed by a histopathologist who was blinded to the HPV status of the participant. The final pathological results were classified as follows: normal or CIN 1, CIN 2, CIN 3 or carcinoma *in situ* (CIS) with either well-differentiated, moderately differentiated or poorly differentiated squamous cell carcinoma or other variants, if applicable.

### 2.4 Statistical analysis

We converted all of the numerical variables into categorical variables before analysis. Gravidity and parity were divided into two groups: less than three and greater than or equal to three. The categorical variables were expressed as frequencies (percentages) for description and analysis by SPSS 23.0 (IBM Corp., Armonk, NY, USA). The associations between age, cervical histopathology, HPV genotype, gravidity, parity and HPV infection patterns were analysed by using the chi-squared test. Univariate and multivariate logistic regression analyses were performed to examine the relationship between multiple HPV infections and cervical lesions, adjusting for age, HPV genotype, and pregnancy. Two-tailed P values less than 0.05.

## 3 Results

### 3.1 Characteristics of the studied patient sample

A total of 6716 female patients with a definite pathological diagnosis were finally included in the analysis. Among them, there were 6124 cases with cervical vascular normality and 592 cases with cervical vascular abnormality. The mean age in 6124 normal cervical vascular patients was significantly higher than that in cervical vascular abnormality patients (41.6 ± 10.9 years old vs. 38.8 ± 10.9 years old, p<0.001). The prevalence of HPV infections was 4284 (70%) in normal cervical vascular patients, while the prevalence of HPV infections was 479 (80%) in cervical vascular abnormality patients. The prevalence of high-grade lesions was 666 (10.9%) in normal patients, while the prevalence of high-grade lesions was 249 (42.1%) in patients with cervical vascular abnormality (P<0.001). The prevalence of cancer was 56(0.9%) in normal cervical vascular patients, while the prevalence of cancer was 72 (12.1%) in patients with cervical vascular abnormality. The prevalence of cervical vascular abnormality across different ages, gravidity, parity, HPV genotype, and histology is shown in **Table 1**.

**Table 1:** Characteristics of the studied patient sample in cervical vascular normality group and cervical vascular abnormality group.

Variables	Total (n = 6716)	Normal (n = 6124)	Abnormal (n = 592)	p
Ages, Mean ± SD	41.4 ± 10.9	41.6 ± 10.9	38.8 ± 10.9	< 0.001
Gravidity, Mean ± SD	2.7 ± 1.6	2.7 ± 1.6	2.5 ± 1.6	0.044
Parity, Mean ± SD	1.5 ± 1.0	1.5 ± 1.0	1.4 ± 1.0	0.016
HPV infections, n (%)				< 0.001
Yes	4763 (70.9)	4284 (70)	479 (80)	
No	1953 (29.1)	1840 (30)	113 (20)	
Cervical cytology results, n (%)				0.809
normal	5211 (77.6)	4754 (77.6)	457 (77.2)	
abnormal	1505 (22.4)	1370 (22.4)	135 (22.8)	
**Histology**				< 0.001
Normal	4307 (64.1)	4089 (66.8)	218 (36.8)	
LSIL	1366 (20.3)	1313 (21.4)	53 (9)	
HSIL	915 (13.6)	666 (10.9)	249 (42.1)	
Cancer	128 (1.9)	56 (0.9)	72 (12.2)	

Normal, cervical vascular normality group; Abnormal, cervical vascular abnormality group; LSIL, low-grade squamous intraepithelial lesion; HSIL, High-grade lesion.

**Figure 3:**
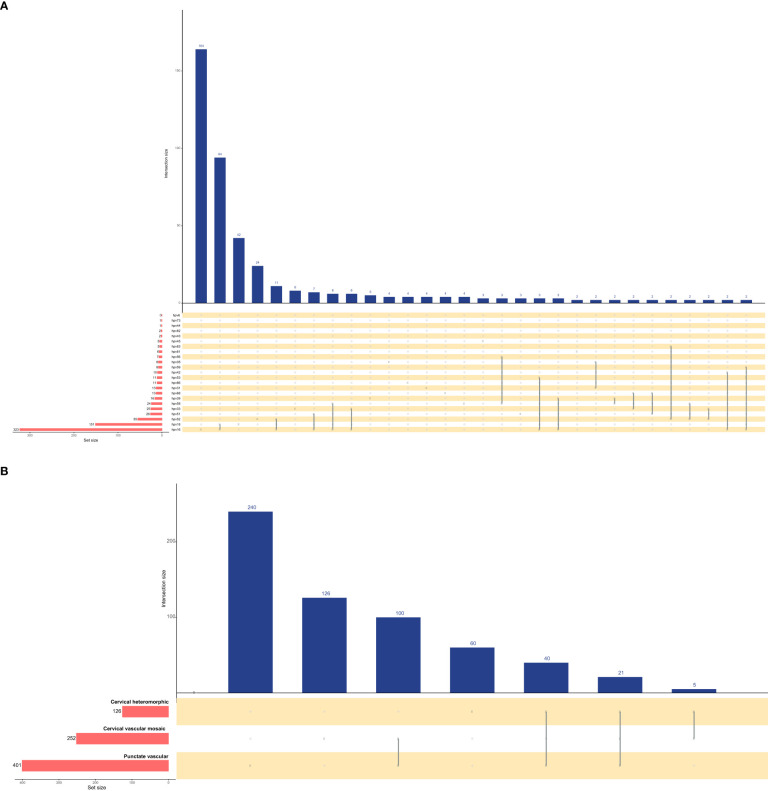
Upset plots of the intersections of HPV genotype **(A)** and cervical vascular abnormality **(B)**. Each row corresponds to a set, and bar charts on the left show the size of the set. Each column corresponds to a possible intersection: the filled-in cells show which set is part of an intersection.

**Figure 3** shows the intersections of the HPV genotype and cervical vascular abnormality as a matrix and reveals contextual information about the set intersections. HPV genotypes 16, 18, 52, 51 and 33 had the most frequent infections, and there was coinfection. Punctation was the most frequent cervical vascular abnormality and was likely to be complicated by cervical vascular mosaic and cervical heteromorphic blood vessels.

### 3.2 Association between different HPV genotypes and punctate vascular features

**Figure 4:**
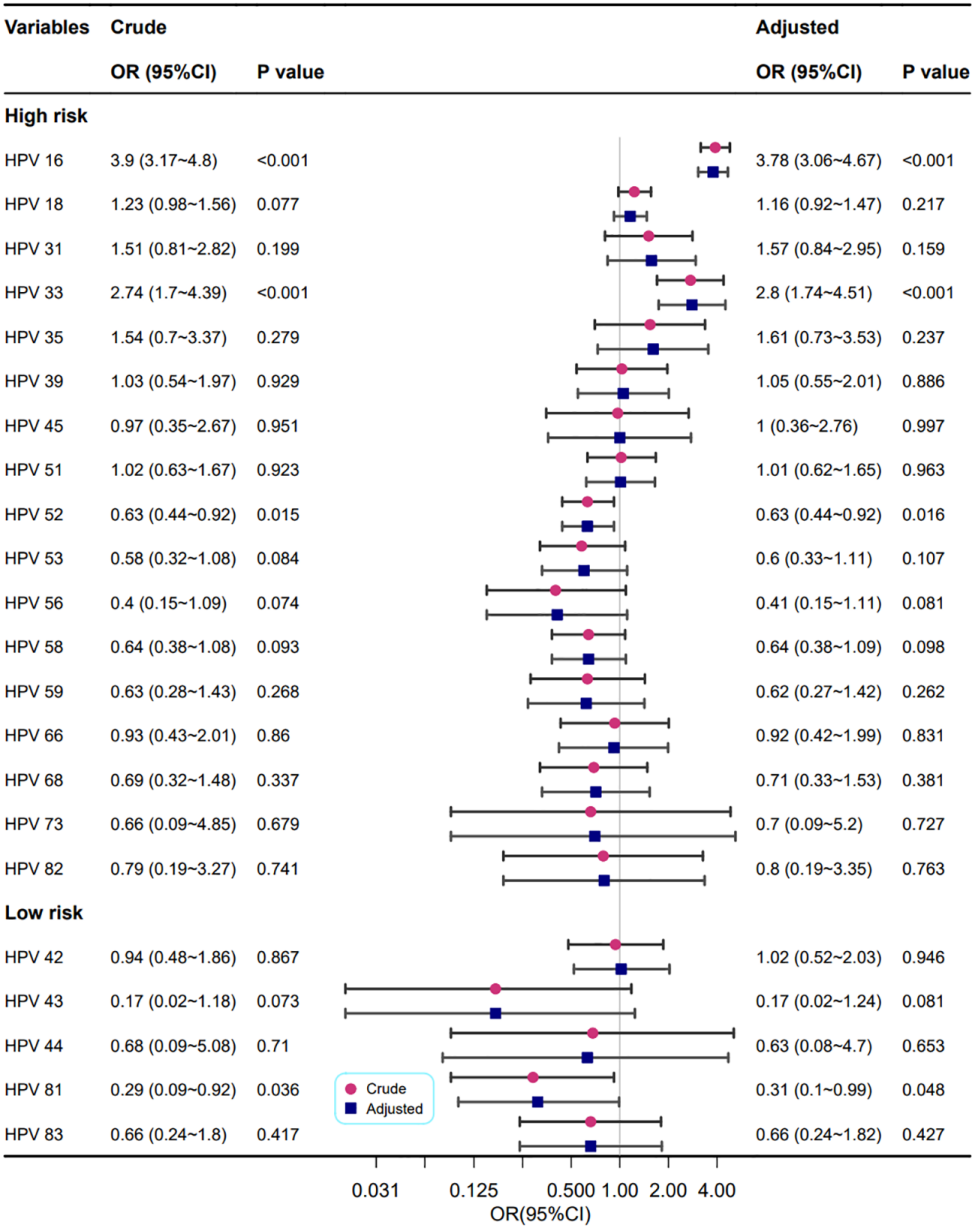
Association between different HPV genotypes and punctate vascular features. Model adjustments for age, gravidity, and parity.

The relationships between different HPV genotypes and punctate vascular features are shown in **Figure 4**. In crude models, the HPV high-risk types of 16, 33, 52 and the HPV low-risk type of 81 were correlated with cervical punctate vascular features. After adjusting for confounding factors, the results consistent with the univariate analysis were obtained, and infection with HPV high-risk types 16 and 33 increased the risk of punctate vascular features by approximately two times or greater (OR=3.78 and 2.80, respectively).

### 3.3 Association between different HPV genotypes and cervical vascular mosaic features

**Figure 5:**
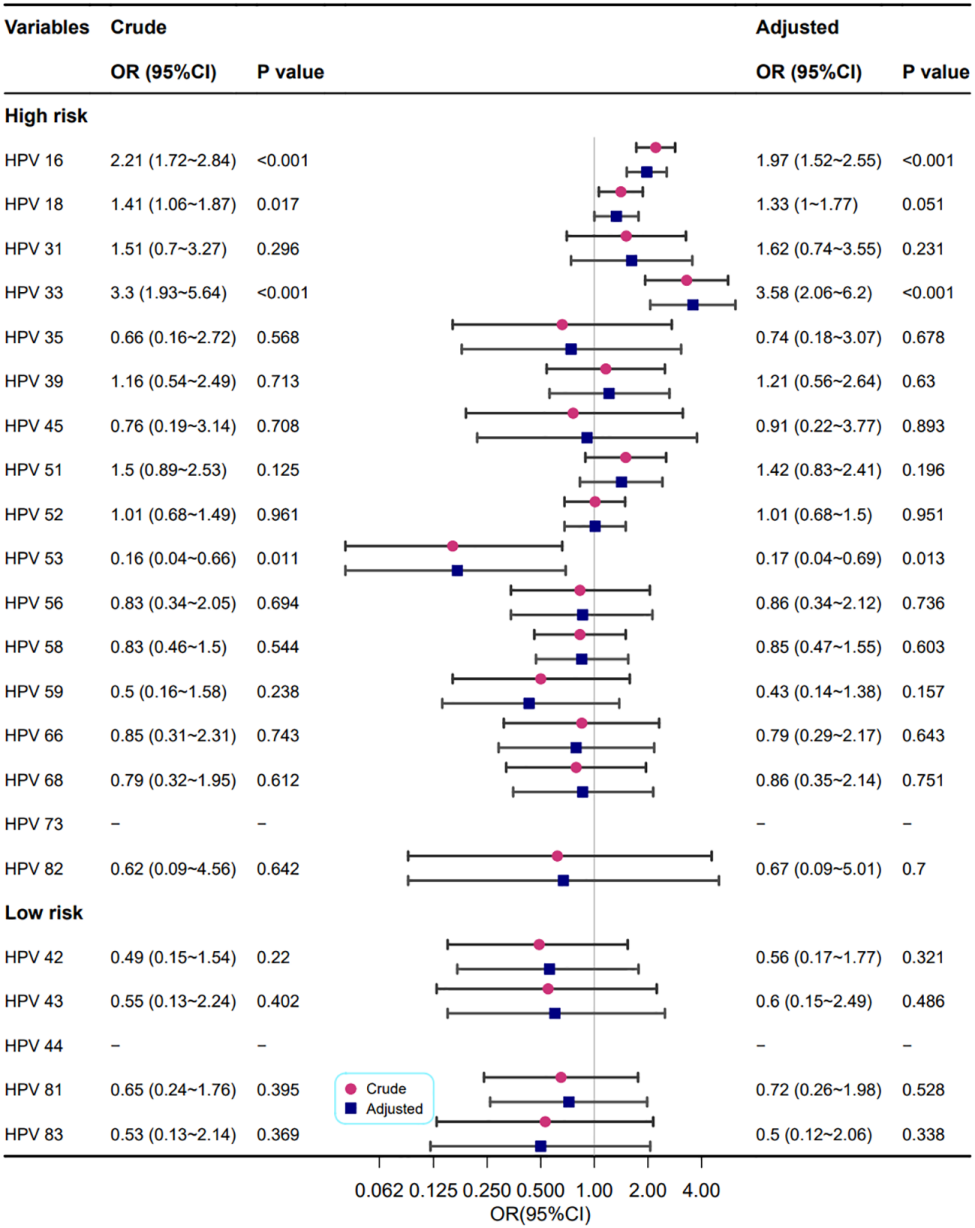
Association between different HPV genotypes and cervical vascular mosaic features. Model adjustments for age, gravidity, and parity.

As shown in **Figure 5**, the relationship between different HPV genotypes and cervical vascular mosaic features was analysed univariately and multivariately. In crude models, the HPV high-risk types 16, 18, 33, and 53 were correlated with abnormal cervical vascular mosaic features. After adjustment for confounding factors, the relationship between HPV genotypes and abnormal cervical vascular mosaic features was mildly changed, and the association of the HPV high-risk type of 18 was no longer statistically significant (P=0.051); however, HPV high-risk types of 16 and 33 increased the risk of cervical vascular mosaic features (OR=1.97 and 3.58, respectively).

### 3.4 Association between different HPV types and cervical heteromorphic blood vessel features

**Figure 6:**
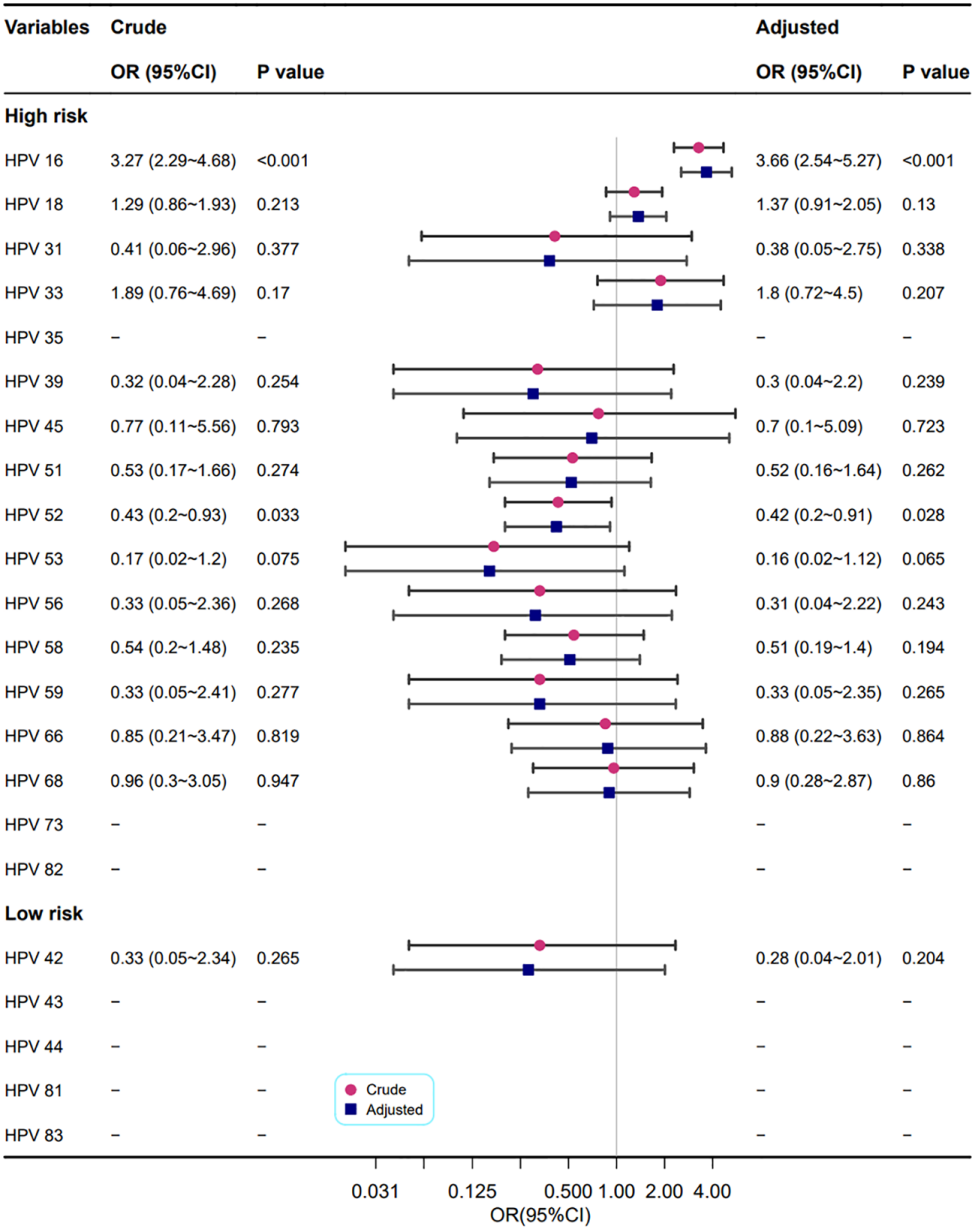
Association between different HPV types and cervical heteromorphic blood vessel features. Model adjustments for age, gravidity, and parity.

**Figure 6** shows the correlation between different HPV genotypes and cervical heteromorphic blood vessel features. In the adjusted models, the HPV high-risk types 16 (OR=3.66, 95% CI: 2.54~5.27, P<0.001) and 52 (OR=0.42, 95% CI: 0.2~0.91, P=0.028) were related to cervical heteromorphic blood vessel features, which is in line with the results of the univariate analysis.

### 3.5 Association between different HPV genotypes and abnormal cervical blood vessel features

**Figure 7:**
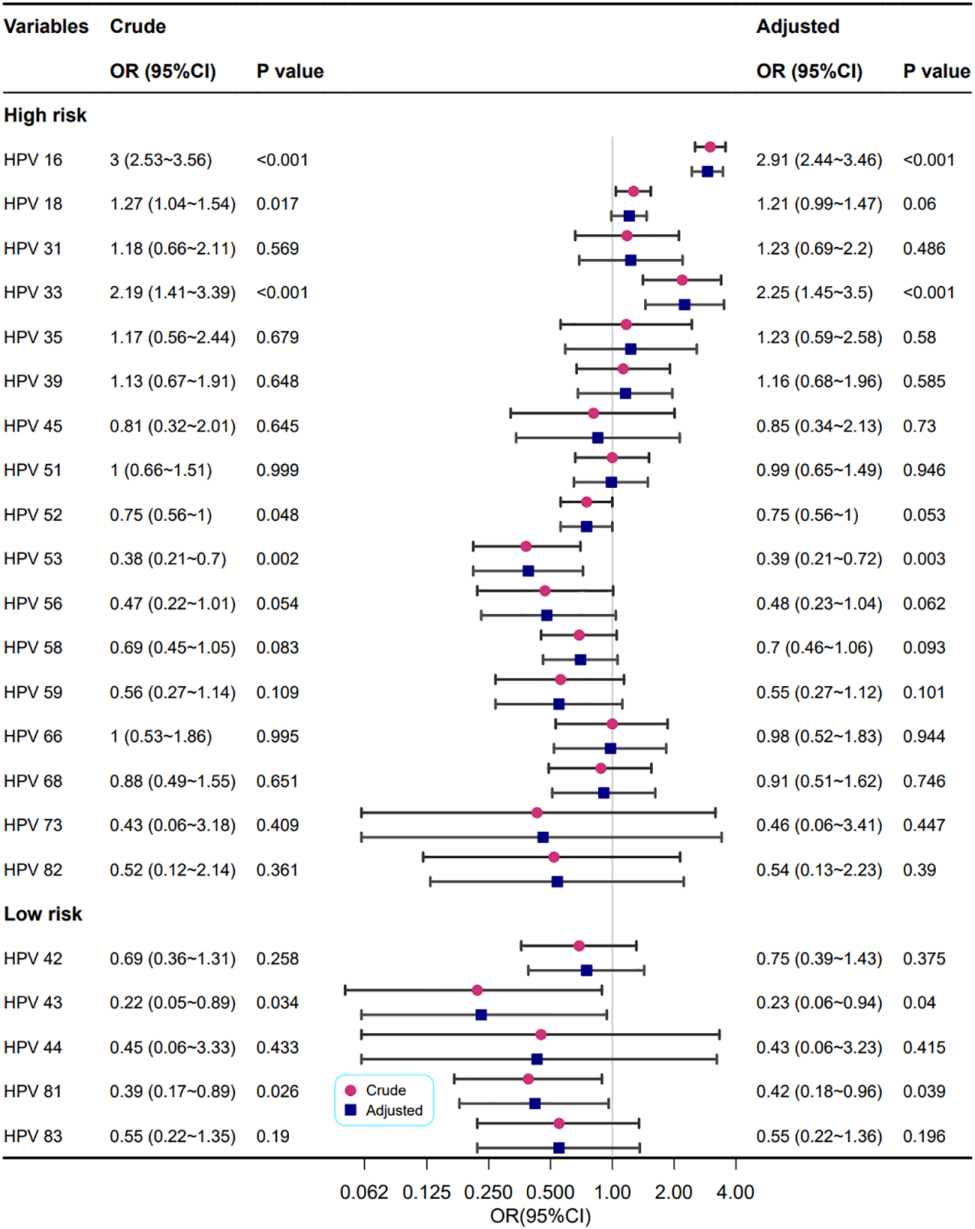
Association between multiple HPV infection and cervical vascular abnormality. Model adjustments for age, gravidity, and parity.

The correlations between different HPV genotypes and abnormal cervical blood vessel features are shown in **Figure 7**. The results showed that infection with HPV types 16 and 33 increased the risk of abnormal cervical blood vessels (OR=2.91, 2.25, respectively), but infection with HPV types 53, 43 and 81 reduced it (OR=0.39, 0.23, and 0.42, respectively).

### 3.6 Association between multiple HPV infection and cervical vascular lesions

The univariate and multivariate logistic regression analyses of multiple HPV infections and abnormal cervical lesions are shown in **Table 2**. Cervical vascular lesions referred to lesions that were demonstrated as punctate vascular, cervical vascular mosaic, cervical heteromorphic blood vessel and abnormal cervical blood vessel. Among the 6716 cases, 2048 were positive for HPV type 16, including 314 cases (15.3%) with abnormal cervical lesions. A total of 122 cases were positive for HPV type 33, including 16 cases (13.1%) with abnormal cervical lesions. The prevalence of HPV 16 and 33 infections was 24 cases, including 9 cases (37.5%) with abnormal cervical lesions. An increased risk of abnormal cervical lesions was observed for HPV 16 and 33 alone or in combined infection compared to the negative group. The risk of abnormal cervical lesions was increased 10-fold by coinfection with HPV 16 and 33 (adjusted OR=10.67,95% CI: 4.54~25.09, P<0.001).

**Table 2:** Univariate and multivariate logistic regression analyses of HPV infection patterns and cervical vascular abnormality.

Variables	Total	Event (%)	Crude		Adjusted	
			OR (95%CI)	P value	OR (95%CI)	P value
Negative	1953	113 (5.8)	1(Ref)		1(Ref)	
HPV16+	2048	314 (15.3)	2.95 (2.36~3.69)	<0.001	2.77 (2.21~3.48)	<0.001
HPV33+	122	16 (13.1)	2.46 (1.41~4.3)	0.002	2.4 (1.37~4.21)	0.002
HPV16+ & HPV33+	24	9 (37.5)	9.77 (4.18~22.81)	<0.001	10.67 (4.54~25.09)	<0.001
Other	2569	140 (5.4)	0.94 (0.73~1.21)	0.626	0.93 (0.72~1.2)	0.554

Model adjustments for age, gravidity, and parity.

### 3.7 Association between multiple HPV infections and lesions above HSIL

The univariate and multivariate logistic regression analyses of multiple HPV infections and abnormal lesions above HSIL are shown in **Table 3**. Among the 6716 cases, 2048 were positive for HPV type 16, including 617 cases (30.1%) with abnormal cervical lesions above the HSIL. A total of 122 cases were positive for HPV type 33, including 43 cases (35.2%) with abnormal cervical lesions above HSIL. The prevalence of HPV 16 and 33 infections was 24 cases, including 15 cases (62.5%) with abnormal cervical lesions above HSIL. HPV 16, 33 or other types of infection were associated with an increased risk of lesions above HSIL compared to the negative group. The risk of lesions above HSIL was up to 26-fold higher in the coinfection with HPV 16 and 33 group than in the negative group (adjusted OR=26.23, 95%CI: 11.23~61.27, P<0.001).

**Table 3:** Univariate and multivariate logistic regression analyses of HPV infection patterns and cervical lesion grades.

Variables	Total	Event (%)	Crude		Adjusted	
			OR (95%CI)	P value	OR (95%CI)	P value
Negative	1953	115 (5.9)	1(Ref)		1(Ref)	
HPV16+	2048	617 (30.1)	6.89 (5.58~8.51)	<0.001	6.9 (5.58~8.53)	<0.001
HPV33+	122	43 (35.2)	8.7 (5.74~13.19)	<0.001	8.7 (5.73~13.19)	<0.001
HPV16+ & HPV33+	24	15 (62.5)	26.64 (11.41~62.17)	<0.001	26.23 (11.23~61.27)	<0.001
Other	2569	253 (9.8)	1.75 (1.39~2.19)	<0.001	1.74 (1.39~2.19)	<0.001

Model adjustments for age, gravidity, and parity.

### 3.8 Sensitivity and additional analyses

To control for multipie tests, we additional use the Benjamini-Hochberg procedure for controlling the false discovery rate. Although p-values of HPV 52+, HPV 53+, HPV 81+ and HPV 43+ became non-statistically significant, for our primary focus on HPV 16+ or HPV 33+ results, the adjusted p-values remained statistically significant (Data not shown).

In addition, the relationship between cervical vascular lesions and cervical lesion grades was shown in **Table 4**. The results showed an increased risk of HSIL and CC in cervical vascular lesions (aOR= 6.79 and 30.23, respectively).

**Table 4:** Multivariate logistic regression analyses of cervical vascular lesions and cervical lesion grades.

Cervical vascular lesions	Total	LSIL	P value	HSIL	P value	Cancer	P value
Normal	6124	1(Ref)		1(Ref)		1(Ref)	
Abnormal	592	0.7 (0.51~ 0.95)	0.021	6.79 (5.55~ 8.3)	<0.001	30.23 (20.43~44.73)	<0.001

Model adjustments for age, gravidity, and parity.

## 4 Discussion

Metastasis and recurrence in patients with angiogenesis-associated CC carry a poor prognosis. Persistent HR-HPV infection leads to angiogenesis; however, how to determine the clinical meaning of local cervical vascular abnormality by colposcopy and its association with HR-HPV remain unknown. The present study assessed the prevalence of the subtype or coinfection of HPV and its correlation with cervical vascular abnormality within a retrospective cross-sectional cohort of 6716 outpatients in Fujian Province in southeastern China. The most prevalent genotype correlated with cervical vascular abnormality was HPV16, followed by HPV33, other than the commonly recognized subtype of 18. Moreover, HPV16 and HPV33 will increase the danger of cervical vascular abnormality and increase the danger of HSIL. Although other types of HPV coinfection also increased the risk of HSIL+, there were not as high as the HPV16 or HPV33 infection. While few studies have found increased risks with coinfection, we found that HPV33 infection increases the risk of cervical lesions above HSIL by 8 times. This is the first study to demonstrate the role of the nine-valent vaccine in preventing cervical lesions caused by HPV33.

Colposcopy is a key instrument for early recognition of cervical lesions, especially vascular abnormalities in the cervix. HR-HPV is the definitive cause of high-grade CIN and CC. However, very few studies have focused on vascular abnormality and local HPV infection. In the 6716 patients included in the analysis, the prevalence of HPV infections was 70% (4284/6716) in normal vascular patients and 80% (479/6716) in patients with cervical vascular abnormality. To the best of our knowledge, the results provide initial direct data on local HPV infections and vascular abnormality by colposcopy. Furthermore, colposcopic vascular abnormality predicted higher cervical lesions, which was consistent with other studies (16). In the current study, the prevalence of high-grade lesions in cervical vascular abnormality patients was higher than that in normal vascular patients (42.1% vs. 10.9%, aOR= 6.79, P<0.001). For CC, the prevalence in patients with cervical vascular abnormality was also obviously higher than that in normal vascular patients (12.2% vs. 0.9%, aOR= 30.23, P<0.001).

Persistent infection is essential for the conversion of a low-grade lesion to a high-grade lesion or cancer, so most HPV infections will not lead to cancer (17). In this study, HPV 16, HPV 33, and coinfection of these two subtypes were significantly related to vascular abnormality in the cervix. However, in contrast to another study showing the equal significance of HPV 18 and HPV 16, we found that vascular abnormality as detected by colposcopy seems to be not significantly associated with HPV 18. Wenbo Long’s research suggests that patients with a high viral load tend to develop more severe cervical lesions, and HPV-16 was the most carcinogenic subtype in Southwestern China, followed by HPV-58 and HPV-33 (17). This observation was slightly different from the prevalence of HPV observed globally (18). Shing Cheng Tan’s research showed that HPV16, HPV18, HPV33, and HPV58 were the most common types of HPV observed across all study specimens (18). One possible reason for this difference could be geographical variability in HPV type distribution (18). The rate of HPV infection was high in our study, and HPV infection was highly correlated with cervical vascular abnormalities.

The biological characteristics of HPV differ with anatomical site. There is a correlation between HPV infection and VEGF surface presence in cervical squamous cell carcinoma. Condyloma acuminatum is a benign hyperplastic disease of the skin and mucosa caused by HPV infection (19). The common viral types causing condyloma acuminatum are HPV 6 and 11, while HPV 16 and 18 are closely related to the occurrence of CC. In the current study, HR-HPV 16 and 33 were recognized as vital factors in vascular abnormalities detected in the cervix by colposcopy. In primary preputial keratinocytes (HFKs), HPV16 E6 and E7 increased the expression of proangiogenic factors such as VEGF and IL-8 (20). At present, the mainstream view is that proteins may participate in the formation of tumour cells in the process of apoptosis and signal transduction and can promote the formation of hypoxia-inducible factors and induce the expression of neovascularization in the early process of tumorigenesis, development and evolution, thus promoting the development of CC. It was also found that the high expression of E6/E7 protein after HPV16 infection mediated the increase in HIF-α and VEGF expression and promoted angiogenesis, thereby promoting the progression of non-small cell lung cancer (21, 22). In CC, high-risk HPV oncoproteins E6 and E7 can promote CC angiogenesis. The HPV-16 oncoprotein promotes the expression of angiogenesis promoters, such as VEGF, to induce angiogenesis (23). This may be the reason why HPV16 promotes cervical vascular abnormalities. The HPV-16 oncoprotein also can promotes the expression of Hypoxia-inducible factor 1α (HIF-1α). HIF-1α is a ubiquitously expressed transcriptional regulator. Activated HIF-1α can relieve the normal regulation of tumor suppressor genes and oncogenes. And then promotes tumor development and progression, which is associated with poor survival prognosis (24). However, the mechanism of action of HPV33 is still unclear and needs further exploration.

High HPV vaccination coverage (95% uptake) may affect the type distribution of HPV infections in the long run (20 years) (25). This phenomenon is called type replacement, characterized by the increased prevalence of nonvaccine-targeted HPV types and a decrease in the prevalence of vaccine-targeted HPV types. However, no type replacement has been observed in a large-scale population with 50% vaccination coverage after 10 years of HPV vaccination (26). In mainland China, the first HPV vaccine, a bivalent vaccine (2vHPV) that targets HPV16/18, was approved in 2016. In 2017, a quadrivalent vaccine (4vHPV) that targeted HPV16/18/6/11 was approved. These two HPV vaccines were also reported to provide cross-protection for HPV 31/33/45/51 (27). The 9-valent HPV vaccine targeting HPV16/18/31/33/45/52/58/6/11 was approved in 2018. However, there are no data regarding HPV vaccine coverage in Fujian Province, and the HPV vaccination statuses of the patients included in this study are unknown. According to two nationwide surveys (28, 29) conducted in 2019 and 2020, the coverage of the HPV vaccine was only 2.6%-3% in females in mainland China. Due to the short time since the HPV vaccine was available on the local market and the low vaccination rate, the prevalence of HPV genotypes and HPV infection patterns in this study are close to the baseline distribution and have not been impacted by HPV vaccination.

In the previous study, female patients who underwent colposcopies/conization following abnormal CC screening results were included, and it’s found that multiple HR-HPV infections do not increase the risk of HSIL+, compared to single HR-HPV infection (30). The previous study did not explore the risk of cervical lesions of different single HPV genotype, but mixed all high-risk HPV together. However, our study only included patients who underwent colposcopy and described cervical blood vessels. When we conducted risk stratification through colposcopy and cervical blood vessels, we found that multiple infections containing HPV 33 would increase the risk of cervical lesions.

The findings are useful for developing HPV vaccination strategies and assessing future HPV vaccine impacts. According to this study, HPV16 and 33 were the most dangerous genotypes that introduced cervical vascular abnormalities. In addition, HPV 16 and HPV33 co-infection may cause more severe cervical vascular lesions than HPV 18. While the 9-valent HPV vaccine is the only currently approved vaccine offering protection against HPV33 in China. Therefore, the 9-valent HPV vaccine could provide the most effective protection against cervical vascular abnormalities.

The major limitation of this study was its retrospective design. It was also limited in that the data collected were from a single institution. The study was a cross-sectional study performed in a single centre and could be affected by selection bias. Moreover, residual confounding, including the number of sexual partners, duration of HPV infection and vaginal microbiota, may continue to influence the result after adjustment for potential confounders. Thus, further large-scale and multicentre prospective cohort studies should be conducted to confirm the results. At last, this study is a clinical study, which the internal mechanism has not been further explored, and we will be further explored in the future.

## 5 Conclusion

HPV16 and HPV33 are the most dangerous HPV genotypes correlated with abnormal vascular patterns. Combined HPV16 and HPV33 infection increases the risk of abnormal vascular patterns. Combined HPV16 and HPV33 infection increases the risk of developing HSIL+.

## Data availability statement

The original contributions presented in the study are included in the article/supplementary material. Further inquiries can be directed to the corresponding authors.

## Ethics statement

The study was conducted according to the guidelines of the Declaration of Helsinki and was approved by the Ethics Committee of Fujian Maternity and Child Health Hospital, Affiliated Hospital of Fujian Medical University (2022YJ002).

## Author contributions

YZ and HL were involved in the design of the study. ZL, XL, QY, YS, YC, and JC carried out data collection and analysis. YZ and HL assisted in drafting the manuscript. YS, HY, and XZ revised the manuscript. All authors contributed to the article and approved the submitted version.
